# Augmented β2-adrenergic signaling dampens the neuroinflammatory response following ischemic stroke and increases stroke size

**DOI:** 10.1186/s12974-019-1506-4

**Published:** 2019-05-28

**Authors:** Kendra J. Lechtenberg, Scott T. Meyer, Janelle B. Doyle, Todd C. Peterson, Marion S. Buckwalter

**Affiliations:** 1 Department of Neurology and Neurological Sciences, Stanford School of Medicine, Palo Alto, Stanford, CA 94305 USA; 2 Department of Neurosurgery, Stanford School of Medicine, Palo Alto, Stanford, CA 94305 USA

**Keywords:** Beta2-adrenergic receptor, Ischemic stroke, Microglia, Macrophage, Neuroinflammation, TNFα, Autonomic, Sympathetic, Infarct

## Abstract

**Background:**

Ischemic stroke provokes a neuroinflammatory response and simultaneously promotes release of epinephrine and norepinephrine by the sympathetic nervous system. This increased sympathetic outflow can act on β2-adrenergic receptors expressed by immune cells such as brain-resident microglia and monocyte-derived macrophages (MDMs), but the effect on post-stroke neuroinflammation is unknown. Thus, we investigated how changes in β2-adrenergic signaling after stroke onset influence the microglia/MDM stroke response, and the specific importance of microglia/MDM β2-adrenergic receptors to post-stroke neuroinflammation.

**Methods:**

To investigate the effects of β2-adrenergic receptor manipulation on post-stroke neuroinflammation, we administered the β2-adrenergic receptor agonist clenbuterol to mice 3 h after the onset of photothrombotic stroke. We immunostained to quantify microglia/MDM numbers and proliferation and to assess morphology and activation 3 days later. We assessed stroke outcomes by measuring infarct volume and functional motor recovery and analyzed gene expression levels of neuroinflammatory molecules. Finally, we evaluated changes in cytokine expression and microglia/MDM response in brains of mice with selective knockout of the β2-adrenergic receptor from microglia and monocyte-lineage cells.

**Results:**

We report that clenbuterol treatment after stroke onset causes enlarged microglia/MDMs and impairs their proliferation, resulting in reduced numbers of these cells in the peri-infarct cortex by 1.7-fold at 3 days after stroke. These changes in microglia/MDMs were associated with increased infarct volume in clenbuterol-treated animals. In mice that had the β2-adrenergic receptor specifically knocked out of microglia/MDMs, there was no change in morphology or numbers of these cells after stroke. However, knockdown of β2-adrenergic receptors in microglia and MDMs resulted in increased expression of TNFα and IL-10 in peri-infarct tissue, while stimulation of β2-adrenergic receptors with clenbuterol had the opposite effect, suppressing TNFα and IL-10 expression.

**Conclusions:**

We identified β2-adrenergic receptor signaling as an important regulator of the neuroimmune response after ischemic stroke. Increased β2-adrenergic signaling after stroke onset generally suppressed the microglia/MDM response, reducing upregulation of both pro- and anti-inflammatory cytokines, and increasing stroke size. In contrast, diminished β2-adrenergic signaling in microglia/MDMs augmented both pro- and anti-inflammatory cytokine expression after stroke. The β2-adrenergic receptor may therefore present a therapeutic target for improving the post-stroke neuroinflammatory and repair process.

## Background

The brain’s inflammatory response to ischemic stroke has significant consequences for secondary neuronal death, effective resolution of the ischemic injury, and stroke outcomes [1, 2]. Thus, defining the endogenous signals that regulate brain inflammation is essential. One such potentially important signal is the sympathetic nervous system. Sympathetic outflow is elevated after stroke in proportion to stroke size, resulting in increased epinephrine and norepinephrine in the blood and cerebrospinal fluid of stroke patients [3–5]. Clinical care of stroke patients also involves amplifying or reducing blood pressure with drugs that act on the adrenergic receptors that bind epinephrine and norepinephrine [6]. This makes it critical to understand how adrenergic signaling affects the immune response to stroke.

The immune effects of adrenergic signaling are primarily transmitted by β2-adrenergic receptors, which are highly expressed on all major immune cell subtypes [7]. β2-adrenergic signaling has context-dependent consequences, in some cases suppressing immune cell activation [5, 8] and in others causing immune cell depletion [9]. Previous studies investigating how β2-adrenergic receptor signaling affects stroke-induced neuronal death and neuroinflammation in the brain focused on manipulation of the receptor prior to stroke only and yielded conflicting results [10–14]. Pre-treating with a selective β2 agonist reduces infarct size in mice [10] and rats [10–12], while global knockout of the β2-adrenergic receptor or pre-treatment with the β2-adrenergic receptor selective antagonist ICI 118,551 also reduces infarct size, improves behavioral outcome, and reduces pro-inflammatory gene expression [13, 14]. These studies did not distinguish whether altered β2-adrenergic signaling directly affects initial stroke size, or if it regulates neuroinflammation and subsequent neuronal loss. These studies also did not examine effects on the immune cells central to the stroke response: microglia and blood monocyte-derived macrophages (MDMs).

Indeed, although it is well-documented that immune cells in the periphery are regulated by the sympathetic nervous system and β2-adrenergic signaling, relatively little is known about the effect of β2-adrenergic signaling on immune responses within the central nervous system. Microglia and MDMs dominate the neuroimmune response in the sub-acute phase after ischemic stroke and therefore have a direct impact on neuronal survival and injury resolution [15, 16]. Both of these cell populations express functional β2-adrenergic receptors [17–19]; in fact, microglia express the β2-adrenergic receptor about ten-fold more highly than any other cell type in the brain [20]. In cultured microglial cells, norepinephrine suppresses LPS-induced NF-κB signaling and production of pro-inflammatory cytokines including iNOS, IL-1β [21–23], tumor necrosis factor-α (TNFα), and IL-6 [18, 24, 25]. Loss of adrenergic signaling in models of neurodegeneration was shown to impair the microglial disease response [25, 26]. However, the effects of β2-adrenergic signaling on the microglial/MDM response to brain injury such as ischemic stroke have not been investigated.

The aim of this study was thus to identify the role of microglial and macrophage β2-adrenergic signaling in modulating neuroinflammation and outcomes in a rodent model of ischemic stroke. We isolated the effects of β2-adrenergic stimulation on post-stroke neuroinflammation by treating mice with the brain-penetrant β2-adrenergic receptor agonist clenbuterol after stroke onset. To specifically investigate the role of microglia/MDM β2-adrenergic signaling on post-stroke neuroinflammation, we induced stroke in a mouse model with selective knockout of the β2-adrenergic receptor from microglia and monocyte-lineage cells. Using these models, we assessed activation, proliferation, and morphology of microglia/MDMs as well as infarct size, expression of inflammatory mediators, and stroke outcomes.

## Methods

### Animals

All animal use was in accordance with protocols approved by the Stanford University Institutional Animal Care and Use Committee. Male C57BL/6J mice (stock number: 000664) and Cx3cr1^CreER^ mice (specific strain name: B6.129P2(Cg)-*Cx3cr1*^*tm2.1(cre/ERT2)Litt*^/WganJ) were purchased from The Jackson Laboratories (Bar Harbor, ME). Adrb2^flox/flox^ mice were obtained from the Gerard Karsenty lab at Columbia University. Mice were housed in a temperature-controlled 12-h light-dark alternating facility, with ad libitum access to food and water. All experiments were performed with 8–11-week-old male mice, except behavioral assessments which were performed in 10-week-old female mice. To generate mice with conditional knockout of the β2-adrenergic receptor from microglia and monocyte lineage cells, we bred Cx3cr1^CreER^ animals with Adrb2^flox/flox^ animals. Experimental Adrb2^cKO^ mice were homozygous for the Adrb2^flox/flox^ allele and heterozygous for the Cx3cr1^CreER^ knock-in allele; littermate control Adrb2^WT^ mice were homozygous for the Adrb2^flox/flox^ allele and homozygous for wild-type Cx3cr1. Both groups were given doses of 0.125 mg/kg tamoxifen in corn oil via oral gavage on three consecutive days to induce knockout of the gene for the β2-adrenergic receptor in Adrb2^cKO^ mice. Stroke surgeries were performed 5 days after the last tamoxifen dose to allow sufficient time for maximal recombination but not enough time for blood monocytes to turn over [27], ensuring that microglia in addition to the majority of blood monocytes would lack the β2-adrenergic receptor at the time of stroke.

### Photothrombotic stroke

The photothrombotic stroke model of cortical ischemic injury was performed based on published protocols [28]. Animals were anesthetized with 2% Isoflurane in 2 L/min 100% oxygen and maintained at 37 °C both during surgery and recovery using a feedback-controlled heating blanket. Mice were injected intraperitoneally (i.p.) with 80 mg/kg Rose Bengal (Sigma #330000 5G) dissolved 10 mg/ml in sterile saline, anesthetized, and placed in a stereotactic frame. Sham mice were given i.p. injections of sterile saline instead of Rose Bengal and underwent the same surgery as the stroke groups. A midline scalp incision was made to expose the skull, and 10 min after Rose Bengal or saline injection, a 1-mm diameter Metal Halide Fiber optic light (Edmundonoptics #56371) was positioned directly over the right motor cortex (0.5 mm anterior and 1.75 mm laterally to the right of Bregma) for exactly 15 min to produce cortical infarction. Mice were concurrently injected with 25 mg/kg cefazolin (VWR #89149-888) and 1 mg/kg of buprenorphine SR (Zoopharm, Windsor, CO) to prevent infection and for pain management, respectively. The scalp incision was resealed using Surgi-lock tissue adhesive (Meridian, Allegan, MI).

### Drug treatment

Clenbuterol (Sigma C5423) was administered to mice 3 h after stroke via subcutaneous pumps with a simultaneous bolus injection to immediately boost the amount of circulating drug in the blood and to allow the drug concentration to reach steady state more quickly. Bolus injections of 1 mg/kg clenbuterol dissolved in sterile 0.9% saline were delivered intraperitoneally using a volume of 0.01 mL per gram of mouse weight. Alzet 1003D drug delivery pumps (Alzet, Cupertino, CA) were filled with 0.000833 g/mL clenbuterol solution to achieve a delivery rate of 1 mg/kg/day and were implanted according to manufacturer instructions 3 h after stroke. Mice in the control group were injected with 0.9% sterile saline and implanted with a pump filled with 0.9% saline. Mice sacrificed at the acute 4-h time point received bolus injections but not subcutaneous pumps. Mice that underwent behavioral assessment were given 1 mg/kg i.p. injections of clenbuterol 3 h post-stroke and subsequently every 24 h for the first week of testing instead of using subcutaneous pump implants, in order to prevent impairment of performance on motor testing. Mice used for histological analysis were injected i.p. with 50 mg/kg bromodeoxyuridine (BrdU: Sigma B5002) dissolved in sterile PBS 24 h prior to sacrifice.

### Immunohistochemistry

All animals were sacrificed at 3 days post-stroke for immunohistochemistry. Mice were sedated with chloral hydrate and perfused with 0.9% NaCl containing 10 U/mL heparin. The brains were collected and drop-fixed in 4% PFA in phosphate buffer for 24 h at 4 °C, then were preserved in 30% sucrose in PBS. A freezing microtome (Microm HM430) was used to collect 40-μm-thick coronal brain sections sequentially into 12 tubes. Brain sections were stored in cryoprotectant medium (30% glycerin, 30% ethylene glycol, and 40% 0.5 M sodium phosphate buffer) at 20 °C until processing. Standard immunohistochemistry procedures were used to stain free-floating sections. Briefly, sections were blocked with 3% donkey (Millipore, #S30-100 mL), or rabbit (Vector, #S-5000) serum for 1 h. Tissue was then incubated at 4 °C overnight in primary antibody: anti-Iba1 (rabbit, 1:1000, Wako 019-19,741), anti-BrdU (rat, 1:5000, Abcam Ab6326), biotinylated anti-NeuN (mouse, 1:500, Millipore MAB377B), or anti-CD68 (rat, 1:1000, BioRad MCA1957S). Iba1 and BrdU were labeled with fluorescent secondary antibodies (donkey anti-rabbit IgG, 1:200, Thermo-Fisher A-21206; donkey anti-rat IgG, 1:200, Jackson ImmunoResearch 712-165-153), mounted onto glass slides, and coverslipped using Vectashield HardSet Mounting Medium (Vector Laboratories, H-1400). For CD68 staining, tissue was incubated for 1 h in secondary antibody (rabbit anti-rat IgG, 1:500, Vector Laboratories BA-4001). For both CD68 and NeuN stains, tissue was treated with Avidin-Biotin Complex solution (Vector, #PK-6100) for 1 h and treated for 5 min with filtered DAB (Sigma, #D5905) solution. Finally, sections were mounted onto glass slides, air-dried overnight, and then coverslipped with Entellan (Electron Microscopy Sciences 14,800). NeuN-labeled slides were rehydrated and stained with Cresyl violet prior to coverslipping.

### Image acquisition and quantification

#### BrdU/Iba1 colocalization

Z-stacks were taken at × 40 magnification using a Leica confocal microscope in the medial peri-infarct region of the cortex, defined as the area equidistant between the stroke border and the midline of the brain, at least one view field above the corpus callosum and with images spaced vertically one view field apart. Two z-stacks per brain section and two brain sections per mouse were used for analysis. Sections were excluded from the analysis if they showed irregular immunostaining, which was either high background immunofluorescence or failure of the BrdU stain to label the subventricular zone, which we used as an internal positive control. Iba1+ cells, BrdU+ cells, and Iba1/BrdU double-positive cells were quantified in the z-stacks using ImageJ software, and percent proliferation was calculated by dividing the total number of Iba1+ BrdU+ double-positive cells by the number of Iba1+ cells. All imaging and quantification and was performed by an experimenter blinded to experimental group.

#### Iba1 fluorescence area

Four z-stacks per brain section in two brain sections per mouse were taken of Iba1-stained cells. Confocal z-stacks of Iba1-stained cells were maximum intensity projected, converted to 8-bit, background-subtracted, and thresholded using the default method in ImageJ software. The percent of the image area covered by Iba1 immunostaining was averaged across images for each individual animal.

#### CD68 immunohistochemistry

Brightfield images were taken at × 20 magnification using a Keyence microscope in the medial and lateral peri-infarct cortex (along the edge of the stroke border). One image was taken per region per tissue section in five sequential sections per animal. Images were converted to 8-bit in ImageJ software and then thresholded using the default method. For each region, the percent of the image area covered by CD68 immunostaining was averaged across images for each individual animal. For qualitative scoring, infarct border images (1 image per brain section, 5 sections per animal) were evaluated by a blinded experimenter. Density of CD68+ cells was given a score of 1 (most sparse), 2 (average density), or 3 (extremely dense). Size of CD68+ cells was given a score of 1 (smallest), 2 (average size), or 3 (largest). Scores per animal were averaged for each measure.

#### Infarct volume analysis of NeuN and cresyl violet immunostains

Slides were scanned using a Silverfast PathScan Enabler IV Slide Scanner. The stroke area, ipsilateral ventricle area, ipsilateral hemisphere total area, contralateral ventricle area, and contralateral hemisphere total area were traced in ImageJ software. In order to account for brain swelling following ischemic injury and to minimize variance, normalized stroke volume was calculated as the stroke area divided by the area of the contralateral brain hemisphere with the contralateral ventricle area subtracted. This value was averaged across the nine sections centered on the maximal area of the stroke for each animal. All quantification was performed by an experimenter blinded to experimental group.

### RNA extraction, reverse transcription, and real-time quantitative PCR

Mice were sacrificed at either an acute (4 h after stroke) or sub-acute (3 days after stroke) time point. Peri-infarct cortical tissue and stroke core was rapidly dissected, flash-frozen using liquid nitrogen, and stored at − 80 °C to prevent RNA degradation. Peri-infarct cortex was defined as the area of cortical tissue within a 2.5 mm radius of the stroke core at the time of dissection. Tissue was homogenized, and RNA was extracted with TRizol reagent (ThermoFisher 15596026). RNA (1.5 μg) was reverse-transcribed into cDNA using the High-Capacity cDNA Reverse Transcription Kit (ThermoFisher 4368814) according to manufacturer’s instructions. We performed qPCR using SYBR Green (Qiagen 204,145) and the QuantStudio 6 Flex Real-Time PCR System. We quantified the expression of the following genes: Adrb2, TNFα, iNOS, IL-10, Ym1, and Mki67 (Table 1). We used Gapdh as a reference gene and calculated relative gene expression using the ddC(t) method [29].

**Table 1:** Primer sequences utilized for RT-qPCR analysis

Gene	GenBank accession number	Gene name	Primer sequence (5′-3′)
Adrb2	NM_007420.3	Adrenergic receptor beta-2	Forward: TCGAGCGACTACAAACCGTC
			Reverse: CCAGAACTCGCACCAGAAGT
TNFα	NM_013693.3	Tumor necrosis factor-alpha	Forward: TAGCCCACGTCGTAGCAAAC
			Reverse: GTCTTTGAGATCCATGCCGTTG
iNOS	NM_001313922.1	Inducible nitric oxide synthase	Forward: TGACGGCAAACATGACTTCAG
			Reverse: GCCATCGGGCATCTGGTA
IL-10	NM_010548.2	Interleukin 10	Forward: CTGGACAACATACTGCTAACCG
			Reverse: GGGCATCACTTCTACCAGGTAA
Ym1	NM_009892.3	Chitinase-like 3	Forward: AGACTTGCGTGACTATGAAGCATT
			Reverse: GCAGGTCCAAACTTCCATCCTC
Mki67	NM_001081117.2	Marker of proliferation Ki-67	Forward: ATCATTGACCGCTCCTTTAGGT
			Reverse: GCTCGCCTTGATGGTTCCT
Gapdh	NM_001289726.1	Glyceraldehyde-3-phosphate dehydrogenase	Forward: ATCATTGACCGCTCCTTTAGGT
			Reverse: GCTCGCCTTGATGGTTCCT

### Behavioral assessment

We tested functional recovery following photothrombotic stroke of the motor cortex using the foot fault task, gridwalk task, and the rotating beam task [30, 31]. We measured missed steps of the contralateral forepaw on the foot fault task, and the distance traveled before falling on the rotating beam task. Mice underwent handling, one habituation day, two pretraining days, and baseline behavioral testing prior to photothrombotic stroke. Behavior was then tested on these tasks on days 1, 3, 7, 14, 21, and 28 following photothrombotic stroke. Animals were placed on the foot fault gridwalk apparatus and were allowed to explore freely for 5 min, and the number of contralateral forelimb faults was recorded. Animals were placed on the rotating beam and the distance covered prior to falling was recorded, for four trials each testing day.

### Epinephrine and norepinephrine ELISA

We measured levels of epinephrine and norepinephrine in plasma of mice 4 and 24 h after photothrombotic stroke or sham surgery using a 2-Catecholamine [Adrenaline-Noradrenaline] Research ELISA kit (Rocky Mountain Diagnostics, Colorado Springs, CO). Briefly, plasma was obtained from whole blood collected via cardiac puncture from deeply anesthetized mice and EDTA and sodium metabisulfite were added to plasma samples at concentrations of 1 mM and 4 mM, respectively. Plasma samples were then diluted 1:2 and were assayed in duplicate according to the manufacturer’s instructions. Three samples were excluded from both the norepinephrine and epinephrine plasma analyses because they were identified to be statistical outliers using published methods [32].

### Blood pressure measurement

Blood pressure was recorded using the non-invasive tail cuff Visitech Systems BP-2000 Blood Pressure Recording System (Apex, NC). Mice were acclimatized to the recording device and procedure for two consecutive days preceding the testing day. Measurements taken on the second acclimatization day were used as baseline blood pressure measurements for each animal. On the testing day, the animals were allowed to adjust to the recording room in their home cages for 1 h, then received a single i.p. injection of either 1 mg/kg clenbuterol or sterile saline. At 4 h and 7 h post-injection, systolic blood pressure, diastolic blood pressure, pulse, and mean arterial blood pressure were measured according to the device manufacturer’s instructions and were processed using BP-2000 software. Fifteen measurements were taken for each animal at each time point, and the value for each animal was calculated by averaging the last 10 measurements taken. Individual measurements that were outliers were identified and removed using published methods [32].

### Statistical analysis

Data was processed using GraphPad Prism 7 software. For comparisons between two groups, a two-tailed Student’s *t* test was used. A Mann-Whitney test with Dunn’s multiple comparisons test was used for data that was not normally distributed. For experiments with more than two groups, a one- or two-way ANOVA test was used with Tukey’s multiple comparisons test for post hoc analysis. All data are presented as mean ± SEM. Experiments were designed using power analyses to determine sample sizes based on expected variances and group differences. All animals were randomized between experimental groups and experimenters were blinded to group assignments.

## Results

### Plasma norepinephrine concentration after photothrombotic stroke

Previous studies have reported an increase in plasma or serum levels of epinephrine and norepinephrine following stroke in humans [3, 33] and in rodent models [5, 9], dependent on stroke severity. We therefore asked if sympathetic catecholamines would be upregulated in plasma in the photothrombotic model of ischemic stroke in mice. There was substantial variability between animals in norepinephrine and epinephrine plasma measurements, and we did not see significant changes in concentrations of either catecholamine at either 4 or 24 h after stroke compared to sham. There did appear to be a slight but non-significant increase in plasma norepinephrine 24 h after stroke (6.11 ± 1.10 ng/mL; mean ± SEM; *n* = 5) compared to sham (3.53 ± 0.68 ng/mL; mean ± SEM; *n* = 5; Dunn’s multiple comparisons test, *p* = 0.4127).

### Increased β2-adrenergic receptor stimulation after ischemic stroke alters microglia/MDM numbers and morphology

**Fig. 1:**
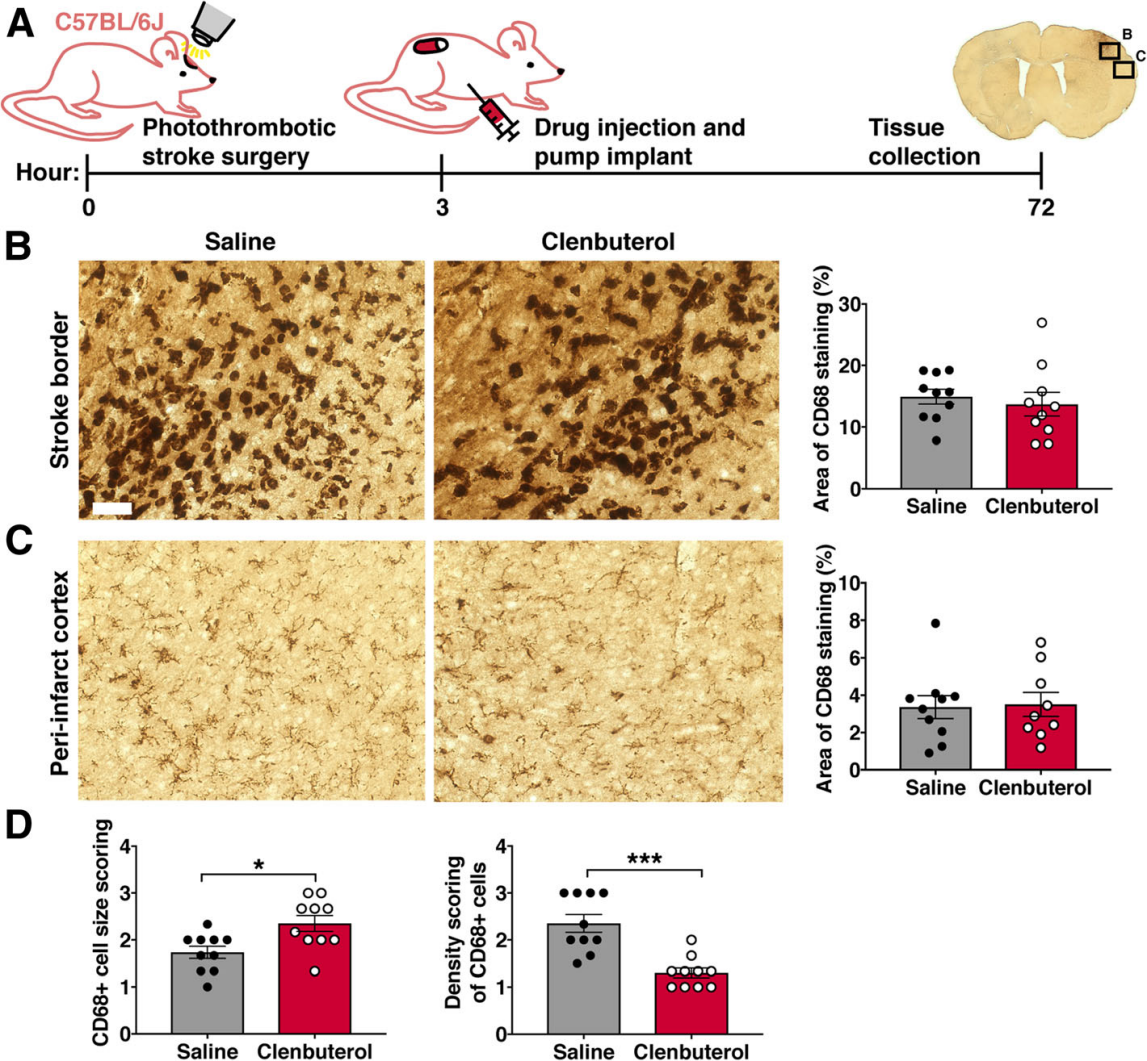
Clenbuterol administration after stroke reduces macrophage density and increases macrophage size. **a** Experimental design. Three hours after photothrombotic cortical stroke, C57BL/6J mice received clenbuterol or saline vehicle control, 1 mg/kg i.p. bolus injection, and 1 mg/kg/day via subcutaneous pump. Mice were sacrificed 3 days post-stroke. **b**, **c** Representative images of CD68-immunopositive cells in the stroke border (**b**) and peri-infarct cortex (**c**) of clenbuterol- or saline-treated mice. Quantification of the percent image area covered by CD68 immunostaining is shown to the right of the photomicrographs, Student’s *t* test. **d** Qualitative scoring of size and density of CD68+ cells in the stroke border, Mann-Whitney test. *n* = 8–10 mice per group. Bars, mean ± SEM; *p < 0.05, ***p < 0.001; scale bar, 50 μm

To investigate how augmenting β2-adrenergic signaling after ischemic stroke influences the microglial and MDM response to stroke, we treated wild-type mice with a brain-penetrant β2-adrenergic receptor-specific agonist, clenbuterol. We administered clenbuterol or saline (vehicle control) 3 h after induction of photothrombotic cortical stroke and collected brain tissue at 3 days post-stroke (Fig. 1a). We first analyzed if clenbuterol treatment after ischemic stroke altered macrophage activation and/or numbers in the cortex by immunostaining for CD68, a marker for activated microglia and macrophages. There was no difference between clenbuterol-treated and saline-treated mice in the percentage area covered by CD68+ macrophages in the stroke border (Fig. 1b) or peri-infarct cortex (Fig. 1c). However, we observed qualitatively that CD68+ cells appeared larger and sparser in the brains of clenbuterol-treated mice. Rating of the size and density of CD68+ cells in the stroke border by a blinded experimenter confirmed that microglia/MDMs were significantly larger and less dense in the brains of clenbuterol-treated mice 3 days after stroke (Fig. 1d), indicating that increased β2-adrenergic stimulation after stroke may induce hypertrophy and reduce cell numbers of microglia and/or MDMs near the infarct.

**Fig. 2:**
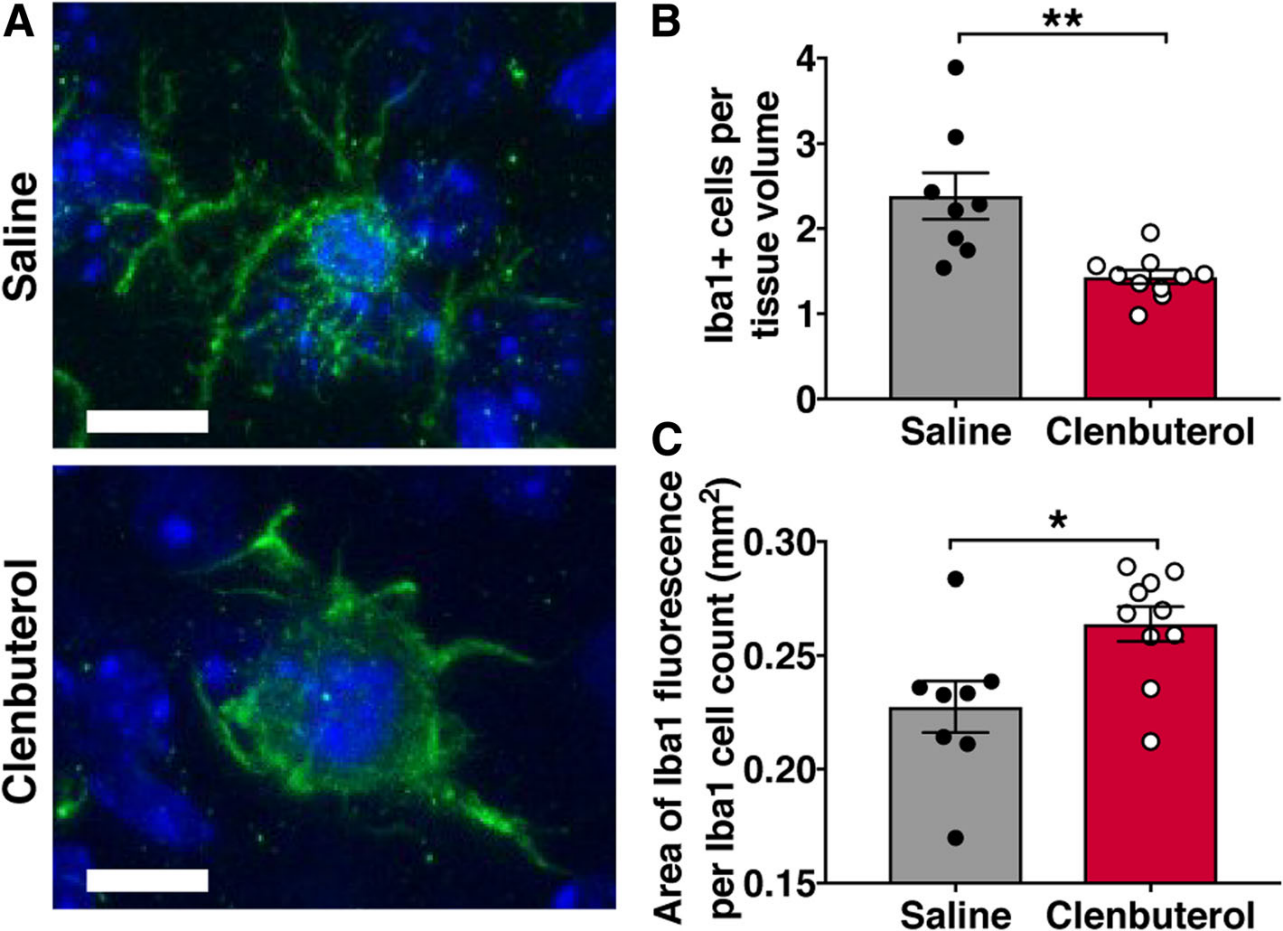
Clenbuterol reduces Iba1+ cell numbers and increases cell size in peri-infarct cortex 3 days after photothrombotic stroke. **a** Representative images of Iba1-immunopositive cells (green) counter-labeled with DAPI (blue) in peri-infarct cortex. **b** Quantification of Iba1+ cells in peri-infarct cortex showed fewer Iba1+ cells in the brains of clenbuterol-treated mice 3 days after stroke. **c** Quantification of the area of Iba1 staining per Iba1+ cell count in peri-infarct cortex of clenbuterol- and saline-treated mice. *n* = 8–10 mice per group. Bars, mean ± SEM; *p < 0.05, **p < 0.01; scale bar, 10 μm

CD68 is a lysosomal protein with high expression in activated microglia and macrophages, but low expression in resting microglia, and thus may not demonstrate cell size accurately. In order to quantify the effect of post-stroke clenbuterol treatment on total microglia/MDM numbers and size, we therefore immunostained for the calcium-binding protein Iba1, which is a cell surface protein on both resting and activated microglial cells as well as MDMs. We counted Iba1-positive cells in confocal z-stacks taken in the peri-infarct cortex, which revealed a 1.7-fold reduction in Iba1+ cell density in the peri-infarct cortex of clenbuterol-treated mice compared to saline-treated mice 3 days after photothrombotic stroke (Fig. 2a, b). Similar to our observations of CD68+ cells, individual Iba1+ cells in stroke cortex of clenbuterol-treated mice appeared hypertrophic. We quantified the area covered by Iba1+ staining per cell and found that it was indeed approximately 14% greater in clenbuterol-treated mice than in saline-treated mice (Fig. 2c).

**Fig. 3:**
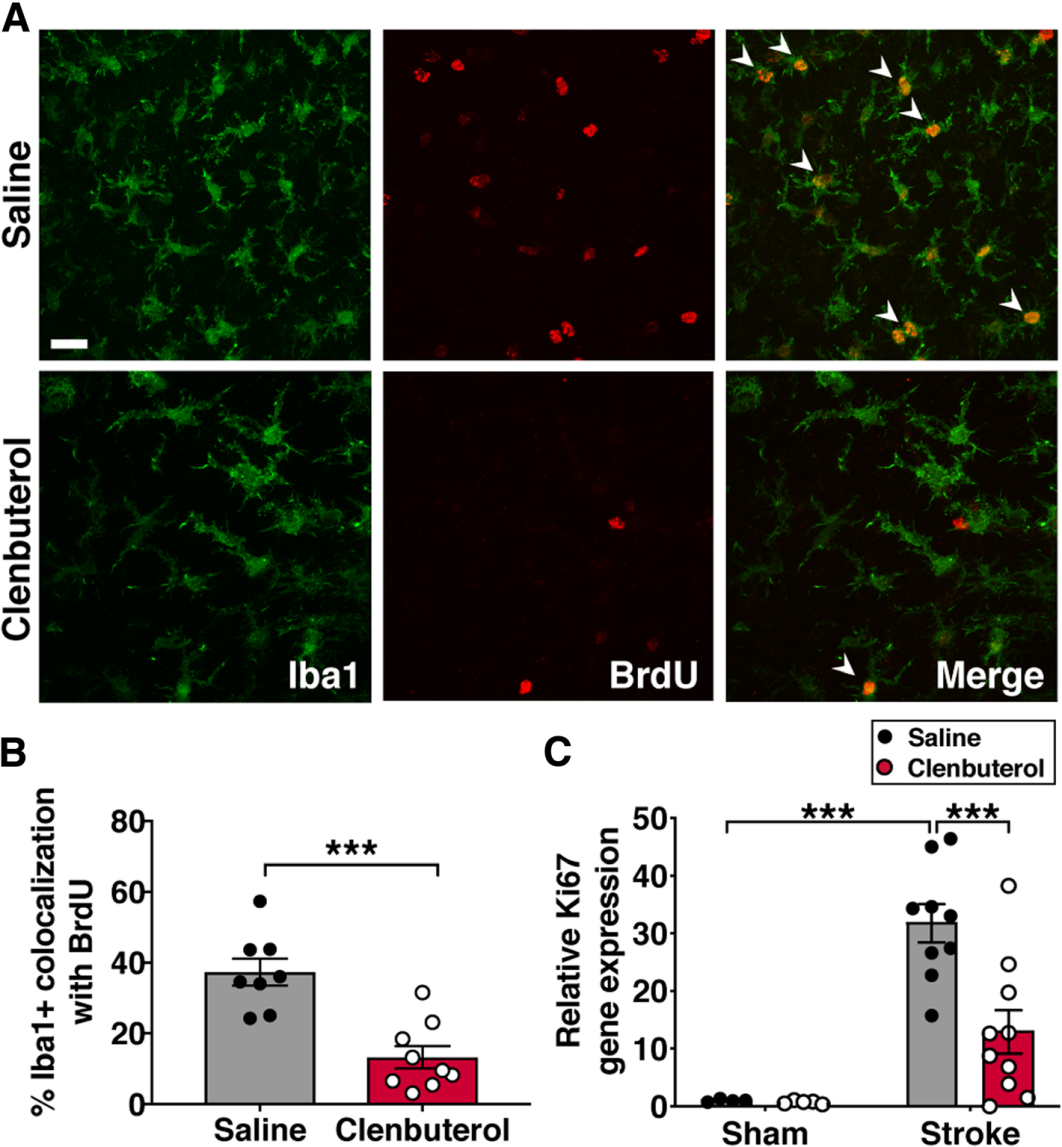
Clenbuterol administration reduces proliferation of Iba1+ cells in peri-infarct cortex 3 days after photothrombotic stroke. **a** Representative images of Iba1 and BrdU colocalization in peri-infarct cortex. **b** Quantification of the percentage of Iba1+ cells co-labeled with BrdU, Student’s *t* test (*n =* 8–10 mice per group). **c** Ki67 gene expression relative to saline-treated sham mice in clenbuterol-treated mice compared to saline-treated mice 3 days after photothrombotic stroke, two-way ANOVA with Tukey’s multiple comparison’s test (*n* = 5–10 mice per group). ***p < 0.001; scale bar, 20 μm

To test if the reduction in numbers of Iba1+ cells in the brains of clenbuterol-treated mice was due to reduced proliferation, we injected mice with the thymidine analog BrdU 24 h prior to sacrifice at 3 days post-stroke and then quantified Iba1+ cells co-labeled with BrdU. The percentage of Iba1+ cells labeled with BrdU was reduced by 2.8-fold in the brains of clenbuterol-treated mice (Fig. 3a, b), suggesting that clenbuterol treatment following stroke reduces microglia/MDM proliferation and numbers surrounding the stroke. Consistent with the reduction in immune cell proliferation, we also observed a decrease in mRNA levels of the non-specific proliferation marker Ki67 in peri-infarct cortex (Fig. 3c). Taken together, these results indicate that increased β2-adrenergic signaling results in impaired proliferation and hypertrophy of microglia/MDMs after stroke.

### Increased β2-adrenergic stimulation after stroke onset causes infarct expansion

**Fig. 4:**
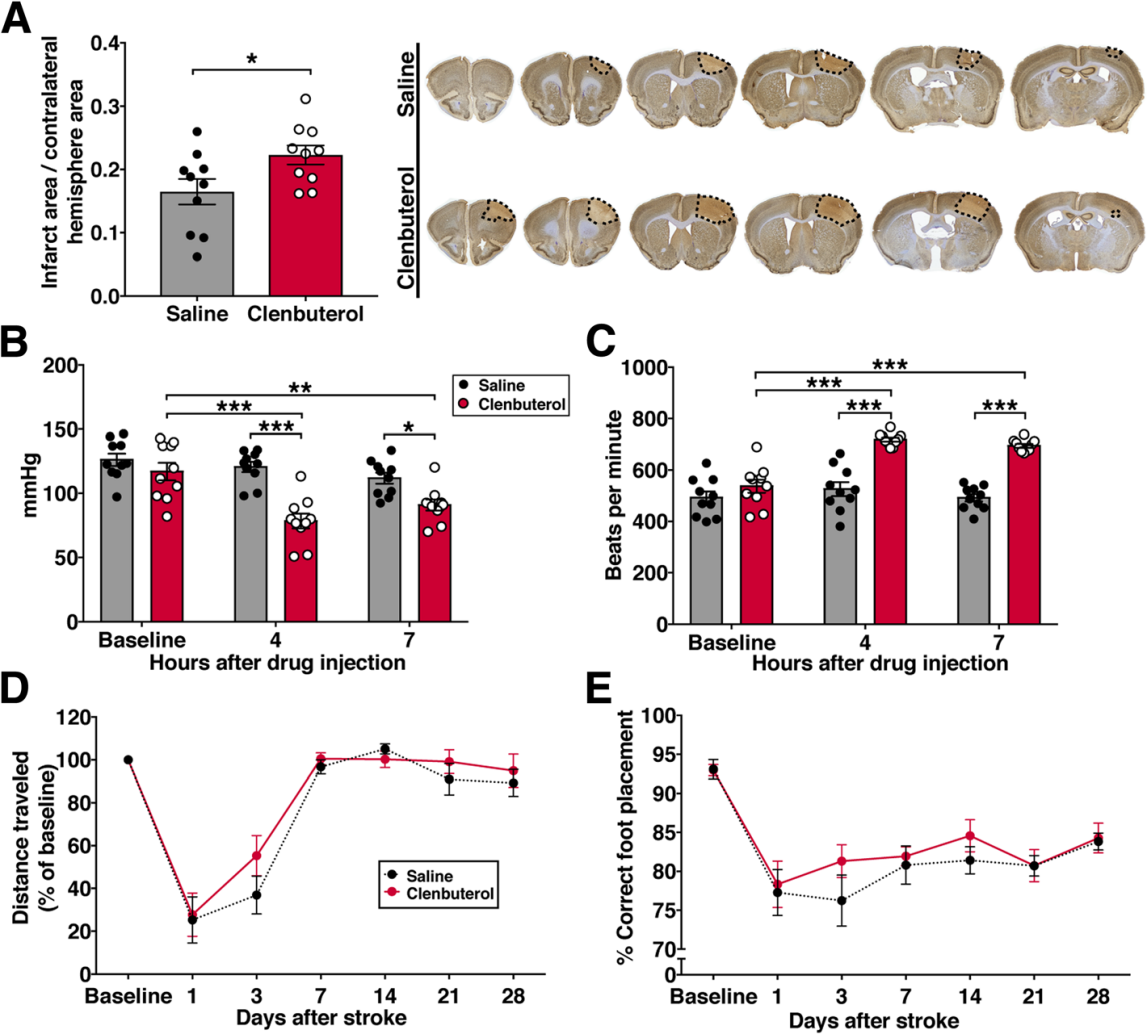
Clenbuterol administration after stroke onset increases infarct size. **a** Quantification of infarct size (normalized to contralateral hemisphere) at 3 days, Student’s *t* test (*n* = 10 mice per group) and representative images of brain sections stained with NeuN and Cresyl Violet showing the anterior-to-posterior spread of the infarcted region at 3 days after stroke in mice treated with saline or clenbuterol. Clenbuterol was given as a 1 mg/kg i.p. bolus injection and 1 mg/kg/day via subcutaneous pump. **b** Quantification of systolic blood pressure and **c** heart rate after a single i.p. injection of 1 mg/kg clenbuterol or saline, two-way repeated measures ANOVA with Sidak’s multiple comparisons test (*n* = 10 mice per group). **d** Performance on the rotating beam task represented as percent of baseline distance traveled (average of four trials). **e** Performance on the foot fault gridwalk task, represented as correctly placed steps as percentage of total steps counted (*n* = 12 mice per group). For behavioral testing, mice were treated with a single 1 mg/kg injection of clenbuterol per day for the first 7 days after stroke. Bars and points, mean ± SEM *p < 0.05, **p < 0.01, ***p < 0.001

Neuroinflammation after stroke affects infarct expansion and cell death, so we asked if the fewer but larger microglia in mice treated with clenbuterol after stroke were associated with changes in infarct size. At 3 days after photothrombotic stroke, clenbuterol-treated animals had 1.4-fold larger infarct volumes compared to saline-treated animals (Fig. 4a). Changes in body weight from baseline as well as spleen weight were not altered by clenbuterol treatment at this time point following stroke (data not shown). Clenbuterol can cause vasodilation by acting on β2-adrenergic receptors expressed by blood vessels, so we evaluated if the dose used for our experiments caused changes in blood pressure in mice. Compared to baseline systolic arterial pressure, we observed a 33% decrease at 4 h and a 22% decrease at 7 h after injection of clenbuterol (1 mg/kg i.p.; Fig. 4b). Diastolic blood pressure and mean arterial blood pressure were similarly reduced (data not shown). As expected, there was a compensatory response in heart rate, which was elevated by 34% at 4 h and 30% at 7 h after clenbuterol treatment (Fig. 4c). We also assessed forelimb motor recovery up to 28 days following photothrombotic stroke but did not observe differences in performance on the rotating beam (Fig. 4d) or the foot fault task (Fig. 4e) with single daily i.p. injections of 1 mg/kg clenbuterol for the first 7 days post-stroke.

### Selective knockout of the β2-adrenergic receptor does not alter post-stroke microglia/MDM morphology

**Fig. 5:**
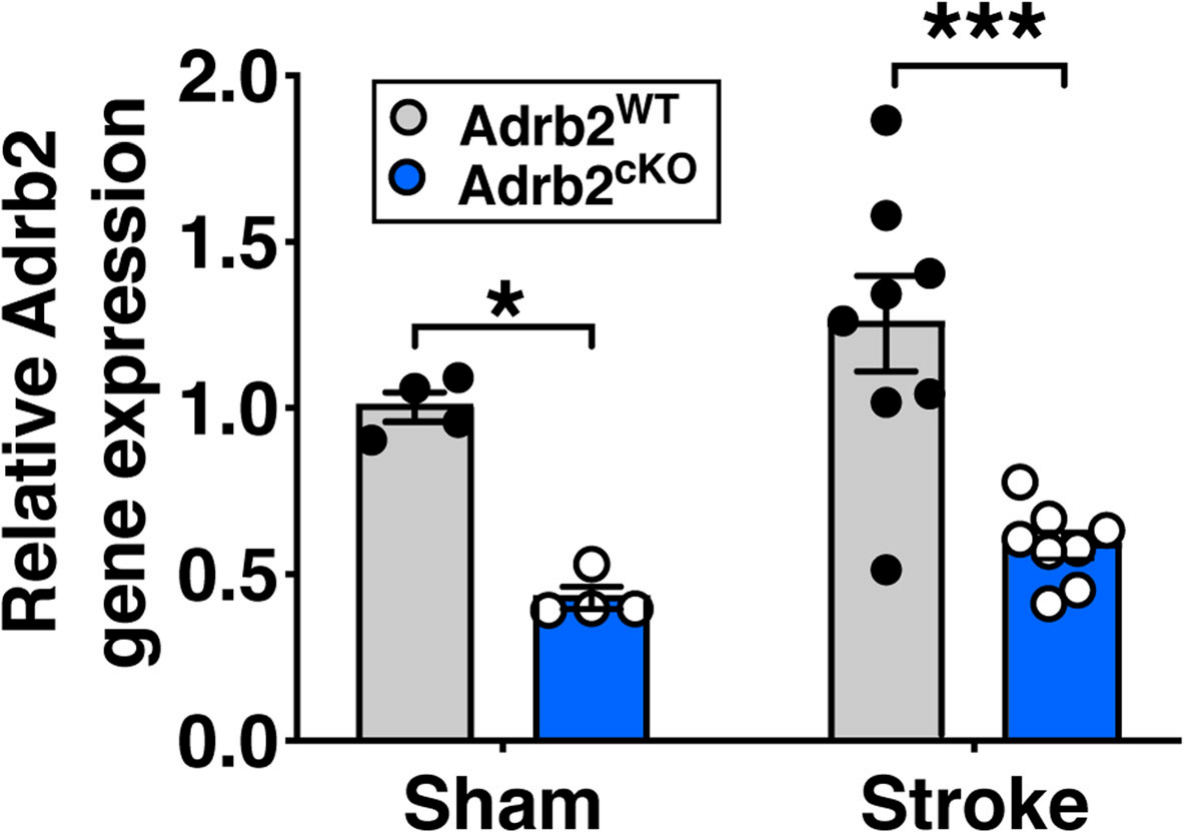
Adrb2 expression is reduced in brain tissue of Adrb2^cKO^ mice. Adrb2 gene expression was calculated using the ddC(t) method relative to expression in wildtype sham mice 3 days after photothrombotic stroke, two-way ANOVA with Tukey’s multiple comparisons test. *n* = 4–8 mice per group. Bars, mean ± SEM; *p < 0.05, ***p < 0.001

Based on our findings that increased β2-adrenergic receptor stimulation dramatically altered morphology and proliferation of brain macrophages after ischemic stroke, we next asked if selectively knocking out the β2-adrenergic receptor from this cell population would alter their stroke response. We generated specific knockouts for Adrb2, the gene for the β2-adrenergic receptor, from cells of the Cx3cr1 lineage (which includes microglia and MDMs). We crossed Cx3cr1^CreER^ mice with Adrb2 floxed mice and induced knockdown by administering tamoxifen, resulting in mice lacking the β2-adrenergic receptor only in Cx3cr1+ cells (Adrb2^cKO^). We confirmed a reduction in Adrb2 mRNA in brain tissue of Adrb2^cKO^ in both sham and stroke conditions (Fig. 5).

**Fig. 6:**
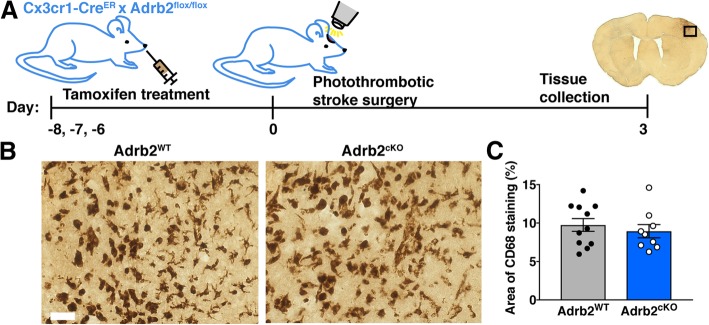
Selective knockout of the β2-adrenergic receptor in microglia/MDMs does not alter cellular morphology after stroke. **a** Experimental design. Cx3cr1^CreER^ x Adrb2^flox/flox^ mice were treated with tamoxifen for 3 days to selectively knock down Adrb2 expression in microglia and MDMs. Five days later, we induced photothrombotic stroke, and mice were sacrificed 3 days after stroke. **b** Representative images of CD68-immunopositive cells in the stroke border of Adrb2^cKO^ or littermate Adrb2^WT^ controls. **c** Quantification of the percent image area covered by CD68 immunostaining showed no difference between Adrb2^cKO^ and Adrb2^WT^ mice, Student’s *t* test. *n* = 9–12 mice per group. Bars, mean ± SEM; scale bar, 50 μm

**Fig. 7:**
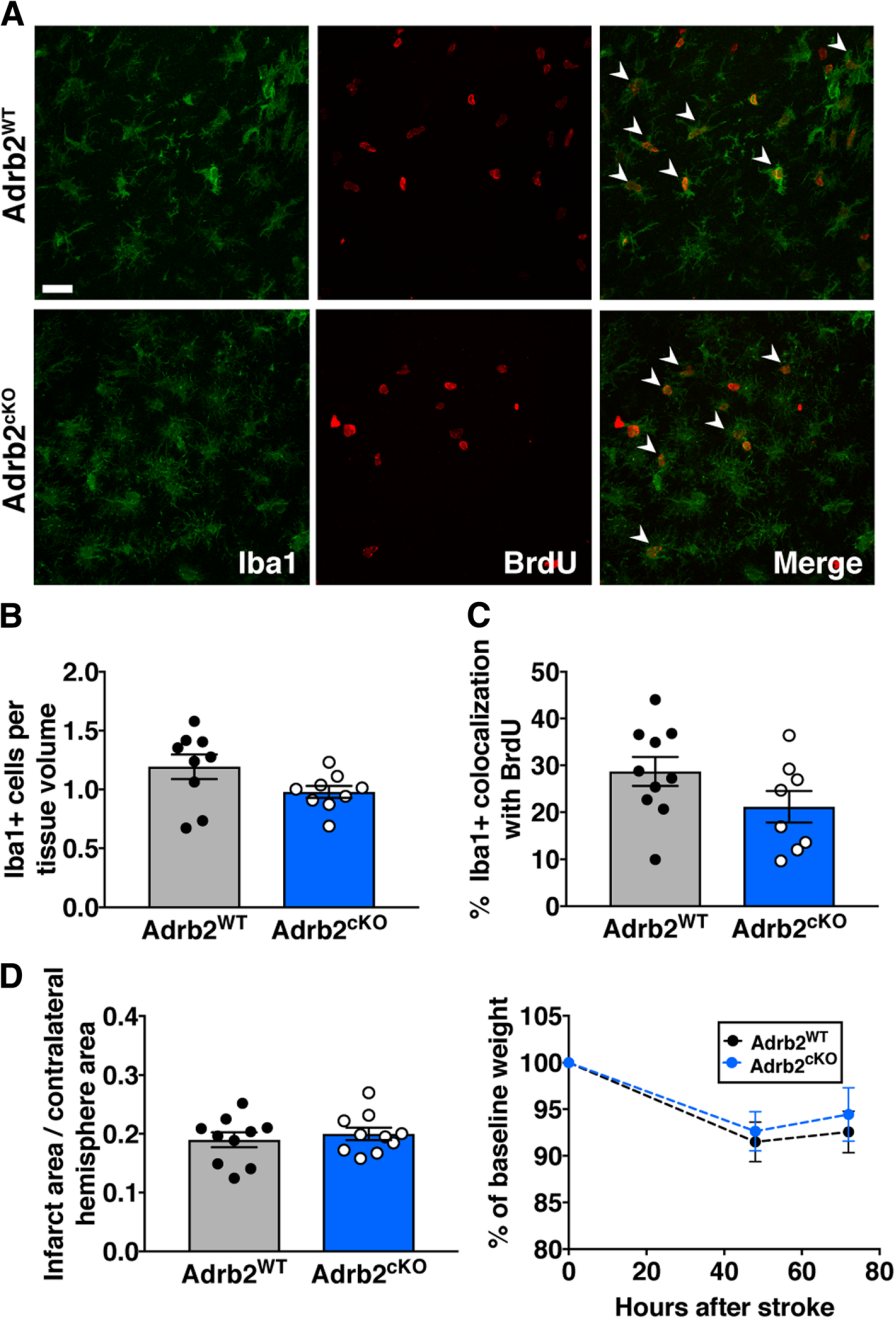
Adrb2^cKO^ mice do not have different Iba1+ cell numbers or infarct size 3 days post-stroke compared to Adrb2^WT^ mice. **a** Representative images of Iba1 and BrdU colocalization in peri-infarct cortex. **b** Quantification of Iba1+ cells in peri-infarct cortex. **c** Quantification of the percentage of Iba1+ cells co-labeled with BrdU. **d** Quantification of infarct size at 3 days, Student’s *t* test (*n* = 8–10 mice per group). Weight loss following stroke was also unchanged. Bars, mean ± SEM; scale bar, 20 μm

Next, we performed photothrombotic stroke in Adrb2^cKO^ and Adrb2^WT^ littermate controls 5 days after tamoxifen administration to allow sufficient time for Cre recombination to knock down expression of the β2-adrenergic receptor from microglia and monocytes (Fig. 6a). To identify changes in microglia and MDM activation in the brains of Adrb2^cKO^ mice, we once again immunostained for CD68 3 days after stroke. The percentage area covered by CD68+ cells in the stroke border or peri-infarct cortex was not different between Adrb2^cKO^ and Adrb2^WT^ mice (Fig. 6b, c). To determine if Adrb2 knockout had more specific effects on microglia/MDM morphology and proliferation after stroke, we also immunostained for Iba1 and BrdU and quantified numbers of Iba1-positive and Iba1/ BrdU-double-positive cells in the peri-infarct cortex of Adrb2^cKO^ and Adrb2^WT^ mice. There was no change in numbers of Iba1+ cells (Fig. 7a, b) or the percentage of Iba1+ cells co-labeled with BrdU (Fig. 7c). In addition, we did not observe differences in infarct size or body weight in Adrb2^cKO^ animals 3 days after photothrombotic stroke (Fig. 7d). There were no differences in these measurements between male and female Adrb2^cKO^ mice (data not shown). Taken together, this suggests that microglia and macrophage-specific Adrb2 deficiency is not itself enough to alter morphology or proliferation of microglia/MDMs or limit infarct size in the subacute period.

### β2-adrenergic signaling regulates expression of inflammatory mediators after stroke

**Fig. 8:**
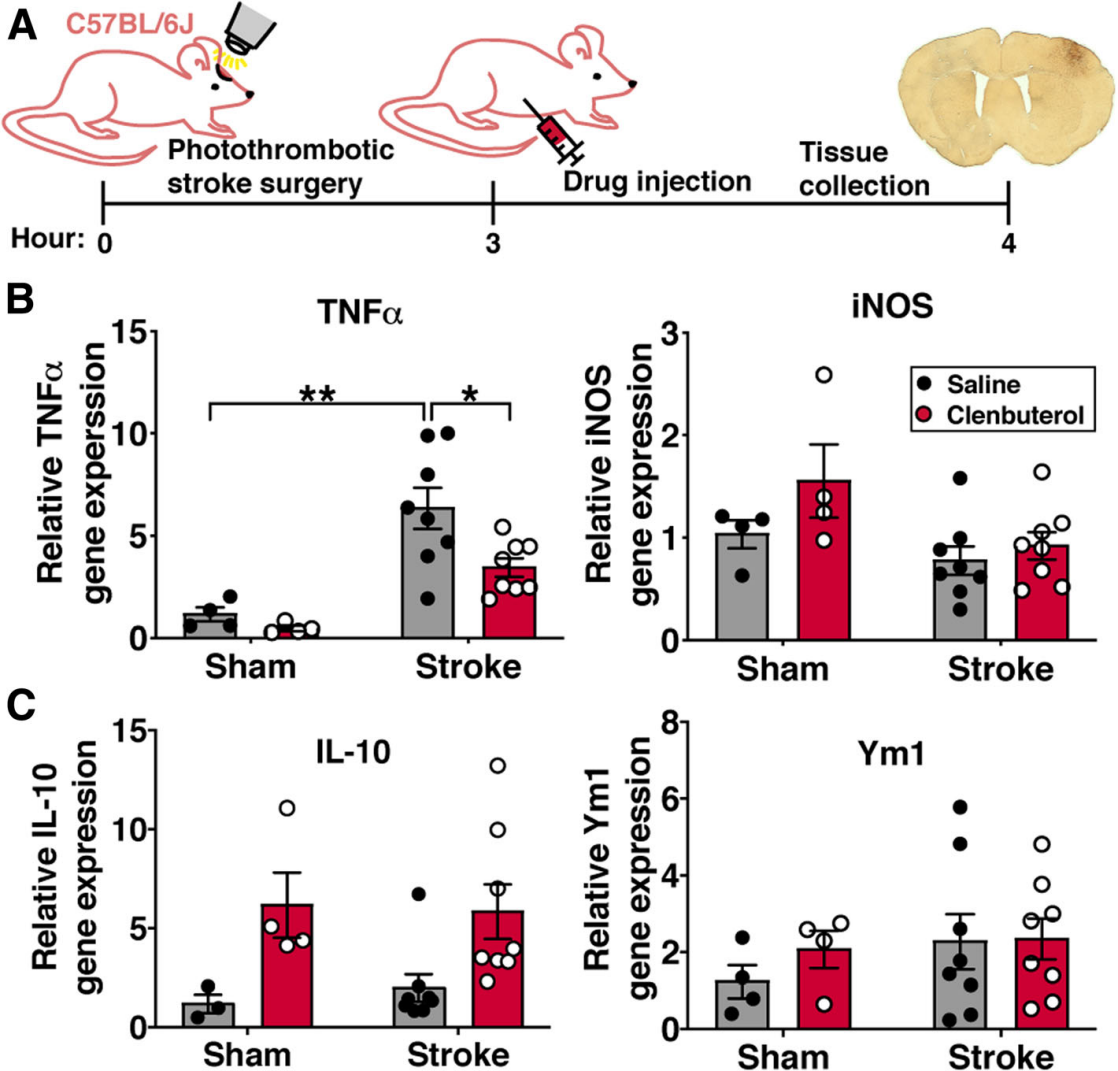
TNFα gene expression at 4 h post-stroke is suppressed by clenbuterol administration after stroke onset. **a** Experimental design. C57BL/6 mice were given 1 mg/kg clenbuterol i.p. 3 h after photothrombotic cortical stroke and peri-infarct cortex was collected 1 h later (at 4 h after stroke) for gene expression analysis. **b** Expression of pro-inflammatory genes TNFα and iNOS were normalized to expression in saline-treated sham mice. **c** Expression of the anti-inflammatory genes IL-10 and Ym1. Two-way ANOVA with Tukey’s multiple comparisons test (*n* = 4–8 mice per group). Bars, mean ± SEM; *p < 0.05, **p < 0.01

We next wanted to ask about the functional effects of β2-adrenergic signaling on microglia and MDMs after stroke. A key function of microglia and MDMs in response to ischemic brain injury is the production of pro-inflammatory signaling molecules such as the cytokine TNFα and inducible nitric oxide synthase (iNOS) [2]. To evaluate whether β2-adrenergic signaling regulates expression of these key inflammatory molecules, we collected peri-infarct cortex at acute (4 h) or subacute (3 days) timepoints after stroke from mice treated with saline or clenbuterol and used quantitative real-time PCR to measure cytokine mRNA levels. At 4 h post-stroke (Fig. 8a), which represents the acute phase of the neuro-inflammatory response and is primarily driven by brain-resident microglia, mRNA expression of TNFα was elevated in peri-infarct brain tissue of both saline- and clenbuterol-treated mice relative to sham mice (Fig. 8b). However, TNFα expression after stroke was 1.8-fold lower in clenbuterol-treated mice compared to saline-treated mice, indicating that increased β2-adrenergic stimulation after stroke suppresses this pro-inflammatory cytokine. TNFα expression has been shown to be regulated by IL-10 [34], so we measured IL-10 mRNA to determine if clenbuterol treatment might be downregulating pro-inflammatory cytokines by upregulating anti-inflammatory gene expression. We observed a trend towards an increase in the anti-inflammatory cytokine IL-10 with clenbuterol treatment in stroke mice (*p =* 0.077) (Fig. 8c). At this early time-point, gene expression of other pro- or anti-inflammatory molecules (iNOS and Ym1) was not affected by stroke or clenbuterol treatment (Fig. 8b, c).

**Fig. 9:**
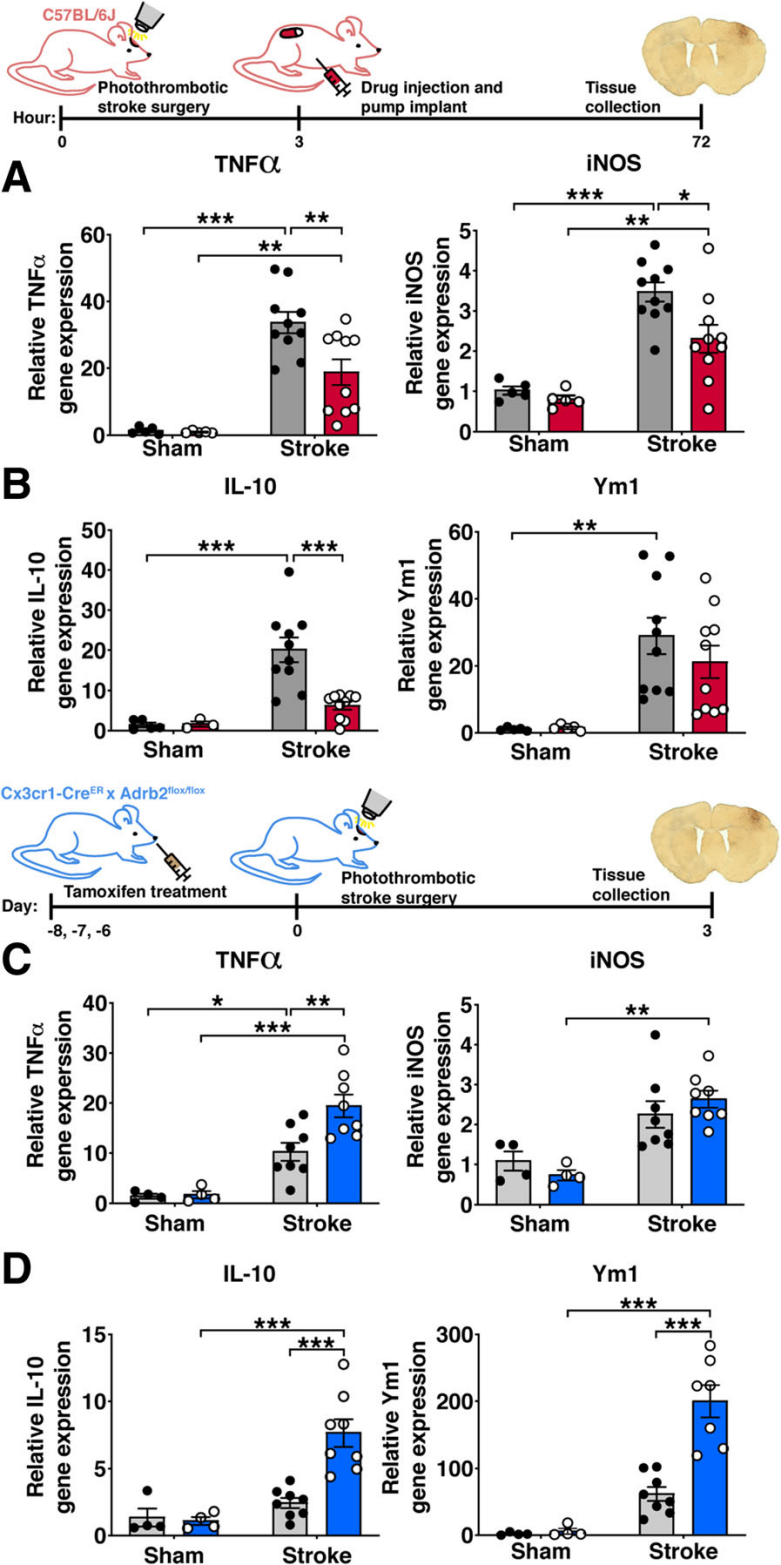
The degree of inflammatory gene expression is regulated by β2-adrenergic receptor signaling 3 days after stroke. Expression of pro-inflammatory genes TNFα and iNOS (**a**) and anti-inflammatory genes IL-10 and Ym1 (**b**) were normalized to expression levels in saline-treated sham mice. There was lower expression of TNFα, iNOS, and IL-10 in clenbuterol-treated mice compared to saline-treated mice after stroke but not sham surgery, two-way ANOVA with Tukey’s multiple comparisons test (*n* = 4–8 mice per group). Expression of genes for TNFα and iNOS (**c**) and IL-10 and Ym1 (**d**) were compared between Adrb2^cKO^ and Adrb2^WT^ mice normalized to expression in sham Adrb2^WT^ mice. Expression of TNFα, IL-10, and Ym1 was higher in Adrb2^cKO^ mice compared to Adrb2^WT^ mice after stroke but not sham surgery, two-way ANOVA with Tukey’s multiple comparisons test (*n =* 4–8 mice per group). Bars, mean ± SEM; *p < 0.05, **p < 0.01, ***p < 0*.*001

In the sub-acute phase of neuroinflammation at 3 days post-stroke, TNFα and IL-10 mRNA expression was substantially elevated in vehicle-treated stroke mice compared to sham mice. As we had seen at 4 h post-stroke, TNFα mRNA expression was 1.8-fold less in peri-infarct cortex of clenbuterol-treated mice relative to saline-treated mice (Fig. 9a). However, in contrast to the trend towards increased IL-10 expression with clenbuterol treatment at 4 h post-stroke, IL-10 mRNA expression was actually reduced by 4.78-fold in peri-infarct cortex of clenbuterol-treated mice relative to saline-treated mice at the 3-day sub-acute time point (Fig. 9b). We hypothesized that the dramatic suppression of TNFα with clenbuterol treatment might be down-regulating the overall brain immune response to stroke at this sub-acute time point, so we also measured mRNA levels of another key pro-inflammatory mediator (iNOS) and an anti-inflammatory molecule (Ym1). Both iNOS and Ym1 were upregulated in response to stroke, and there was 1.5-fold less iNOS mRNA expression in peri-infarct cortex of clenbuterol-treated mice relative to saline-treated mice (Fig. 9a). These results suggest that clenbuterol treatment rapidly suppresses TNFα expression after stroke and prolonged clenbuterol treatment after stroke onset subsequently results in a suppression of both pro- and anti-inflammatory responses.

We next measured overall gene expression of pro- and anti-inflammatory signaling molecules in the peri-infarct cortex of Adrb2^cKO^ mice and Adrb2^WT^ controls 3 days after stroke. We hypothesized that we would not observe changes in cytokine gene expression, since we did not observe differences in Iba1+ cell numbers and morphology or infarct size in these mice. Unexpectedly, we found a 1.89-fold increase in TNFα mRNA in the peri-infarct cortex of Adrb2^cKO^ mice compared to Adrb2^WT^ mice (Fig. 9c), and a 3.15-fold increase in IL-10 mRNA compared to Adrb2^WT^ controls (Fig. 9d). Unlike with clenbuterol treatment, Adrb2 knockout did not change gene expression of iNOS (Fig. 9c). Expression of the anti-inflammatory marker Ym1 was also 3.24-fold higher in the brains of Adrb2^cKO^ mice after stroke relative to Adrb2^WT^ controls (Fig. 9d). Ym1 is predominantly expressed by microglia and macrophages in the brain, so it appears that Adrb2 knockout from these cell types upregulates genes associated with both pro- and anti-inflammatory responses. These findings show that globally increasing β2-adrenergic receptor stimulation with clenbuterol treatment dampens stroke-induced expression of both pro- and anti-inflammatory mediators in the post-stroke brain, whereas selective knockout of Adrb2 from Cx3cr1-lineage cells is sufficient to increase post-stroke expression of both pro- and anti-inflammatory genes.

## Discussion

We show here that increasing β2-adrenergic stimulation after stroke onset causes enlarged morphology of microglia/MDMs surrounding the stroke, impairs proliferation of these cells, and reduces expression of pro-inflammatory TNFα early, followed by a later reduction in both pro- and anti-inflammatory cytokines. Augmented β2-adrenergic signaling after stroke resulted in decreased blood pressure, increased heart rate, and increased infarct volume. Furthermore, we report that selective knockout of the β2-adrenergic receptor causes opposite changes in the overall cytokine response in the post-stroke brain but does not affect microglial proliferation or morphology. Taken together, this indicates that β2-adrenergic signaling is an important regulator of the microglia/MDM response to ischemic brain injury and that increasing it after stroke is detrimental.

There is a growing interest in understanding the impact of drugs targeting the sympathetic nervous system on stroke outcomes. Analyses of clinical stroke populations report that more than 50% of patients had prolonged use of β-blockers prior to experiencing stroke, some of which act on the β2-receptor [35, 36]. Also, after stroke, patients may receive therapy with pressors to increase their blood pressure, and some act on the β2-receptor (e.g., norepinephrine and epinephrine), while others do not (e.g., neosynephrine) [6]. Clinically, the choice of adrenergic agent does not take into account direct or indirect effects on neuroinflammation, because these effects are unknown. Thus, it is critically important to understand how the sympathetic nervous system regulates the pathophysiology of stroke both in the periphery and in the central nervous system. β-blockers are also being investigated to prevent or treat post-stroke infection, which is believed to be caused in part by immunosuppression due to an increased outflow of the sympathetic nervous system. Some retrospective studies of β-blocker use in stroke patients found reduced infection and improved outcomes [37]. However, other similar studies failed to find improvements in infection rates and mortality with β-blocker use [36, 38, 39]. Further work will be required to understand whether post-stroke neuroinflammation in humans is analogous to neuroinflammation in mice. Our results imply that both non-selective β-blocker and pressor use in humans may be influencing stroke outcome.

The goal of our study was to directly assess the effect of β2-adrenergic signaling on post-stroke neuroinflammation in the central nervous system by treating mice with the β2-adrenergic receptor agonist clenbuterol after the ischemic event had already occurred. We used a stroke model that produces small-to-moderately-sized strokes that did not induce a significant increase in plasma norepinephrine and epinephrine. These results are in line with earlier findings from both human and animal studies that stroke size correlates with sympathetic response [3–5] and reflect that this model may not induce strokes that are large enough to increase plasma catecholamines. Our results demonstrate that pharmacologically supplementing β2-adrenergic signaling after photothrombotic stroke suppresses the microglia/MDM response and the expression of inflammatory mediators. The pro-inflammatory cytokine TNFα was strongly reduced early after stroke with clenbuterol treatment and prolonged clenbuterol treatment after stroke led to a suppression of both pro- and anti-inflammatory mediators. These findings indicate an overall downregulation of the neuroimmune response with increased β2-adrenergic signaling. The effects on cytokine expression are mediated by microglia and MDMs, as cell-specific knockout of the β2-adrenergic receptor led to increased cytokine expression. As noted above, drugs regulating adrenergic signaling typically affect blood pressure by acting on adrenergic receptors expressed in the heart and in blood vessels [7]. We observed a reduction in rodent blood pressure and a compensatory increase in heart rate with the dose of clenbuterol used for this study. The neuroimmune effects of increased β2-adrenergic stimulation after stroke may therefore be due to direct binding to β2-adrenergic receptors expressed by immune cells as well as indirect effects mediated by changes in blood pressure. Further experimentation would be needed to determine if clenbuterol reduces cerebral perfusion specifically in the context of ischemic stroke.

The finding that the β2-adrenergic receptor agonist clenbuterol leads to infarct expansion is in contrast to previous reports that rats or mice treated with clenbuterol 3–5 h prior to transient or permanent middle cerebral artery occlusion (MCAO) have reduced infarct volumes at 7 days post-stroke [10–12]. The disparities in outcomes between our observations and these previous studies may be explained by differences in stroke model, drug dose, or experimental timelines. For instance, particularly high doses of clenbuterol administered before stroke in rats increased ischemic damage, which the authors suggested was due to a dramatic reduction in blood pressure and therefore cerebral perfusion [12]. Lower doses of clenbuterol might confer neuroprotective effects without reducing blood pressure. However, pretreatment with clenbuterol at doses that reduced blood pressure was neuroprotective in another rodent stroke study [40], so it is unclear if the beneficial or detrimental effects of clenbuterol can be attributed to blood pressure changes alone. Another possibility is that clenbuterol targets different mechanisms in stroke pathophysiology depending on the timing of administration. Thus, clenbuterol treatment before stroke onset could mitigate the severity of the initial ischemic event, whereas clenbuterol treatment after stroke could reduce cerebral blood flow and suppress the normal neuroimmune response, resulting in exacerbation of the injury.

Interestingly, blocking β2-adrenergic signaling is also reported to be neuroprotective in stroke models. For example, infarct size 24 h after transient MCAO is reduced in global β2-adrenergic receptor knockout mice or mice treated with the β2-adrenergic receptor antagonist ICI 118,551 prior to stroke [13, 14]. Pre-treatment with the non-specific β-adrenergic receptor antagonist propranolol also reduces stroke size 3 days after MCAO in rats [41]. These three studies taken with our results suggest that blocking β2-adrenergic receptor activity before stroke onset is neuroprotective, whereas increasing β2-adrenergic receptor signaling after stroke is detrimental.

Our data show for the first time that β2-adrenergic signaling specifically regulates the microglial and MDM response to ischemic stroke. We observed that clenbuterol treatment after stroke onset induced enlarged morphology of microglia/MDMs in the peri-infarct cortex and caused a dramatic reduction in microglia/MDM numbers and proliferation. These findings are in agreement with previous reports of the effects of β2-adrenergic stimulation on microglia in vitro. In an acute brain slice model, stimulation of microglia with norepinephrine causes retraction of microglial processes and a shift to an ameboid morphology, dependent on β2-adrenergic receptors [42]. Furthermore, stimulation of cultured rat microglia with β2-adrenergic receptor agonists but not agonists for other adrenergic receptor subtypes suppresses microglial proliferation, possibly by elevating intracellular levels of cAMP [43]. β2-adrenergic stimulation also suppresses the proliferation of other immune cell subtypes in vivo, such as group 2 innate lymphoid cells [44]. We assessed microglia/MDM numbers, activation, and proliferation in mice with Adrb2 specifically knocked out of monocyte-lineage cells in order to determine the effects of β2-adrenergic receptor knockout on how these cells respond to stroke. We surprisingly did not see an increase in proliferation of microglia/MDMs in Adrb2^cKO^ mice, suggesting that cell-intrinsic β2-adrenergic signaling may not regulate the effects of clenbuterol specifically on proliferation, and/or that there are other signals influencing proliferation in the absence of β2-signaling. One possibility is that the effect of clenbuterol treatment on blood pressure and/or heart rate indirectly suppresses microglia/MDM proliferation or impairs MDM trafficking into the injured brain.

In addition to proliferation, a key function of microglia and MDMs in post-stroke inflammation is upregulation of inflammatory signaling molecules, such as TNFα and iNOS. In clenbuterol-treated mice, post-stroke expression of these genes was reduced. Notably, we report higher expression of TNFα in Adrb2^cKO^ animals after stroke, in which the β2-adrenergic receptor is specifically knocked out of microglia/MDMs. These results suggest that the changes in inflammatory gene expression with clenbuterol are directly mediated by intrinsic microglia/MDM β2-adrenergic signaling rather than by indirect effects such as blood pressure. Our findings are in agreement with substantial previous literature that β2-adrenergic stimulation is generally anti-inflammatory. For example, clenbuterol treatment reduces gene expression of TNFα and NFκb signaling in the brain after systemic LPS treatment [45]. The opposite effect occurs in rodent models of Alzheimer’s disease, where disproportionate death of noradrenergic neurons causes increased expression of TNFα and iNOS, which can be reduced with norepinephrine administration [26]. In the context of neurodegeneration, it appears that loss of adrenergic signaling and the resulting increase in neuroinflammation exacerbates neuronal loss and speeds disease progression [25, 26, 46]. However, in our stroke model, increasing adrenergic signaling and reducing neuroinflammation was associated with increased stroke size. This finding may support the concept that a robust early neuroinflammatory response is actually beneficial for stroke outcomes, possibly by promoting clearance of harmful debris and dead cells, recruiting immune cells from the periphery, and forming a border around the stroke. Other studies also support this idea; for instance, depleting microglia from the brain before ischemic stroke or preventing MDM influx into the brain results in larger infarcts and worse outcomes [15, 47, 48]. Alternatively, it may be that blood pressure reduction by clenbuterol was responsible for the increased stroke size independent of clenbuterol-induced changes in neuroinflammation.

At 4 h post-stroke with clenbuterol treatment, we observed a significant decrease in TNFα gene expression in the stroke condition accompanied by a trend towards an increase in IL-10 gene expression. At 3 days after stroke, both cytokines are decreased by clenbuterol treatment, and both are increased in the Adrb2^cKO^ mice. Although the change in IL-10 with clenbuterol at 4 h after stroke is not significant, this data is consistent with previous reports that stimulation of β2-adrenergic receptors with clenbuterol or norepinephrine induces IL-10 expression and suppresses TNFα expression [18, 24, 49–51]. The fact that 3 days later both are suppressed by clenbuterol likely results from a feedback loop between these two cytokines. Upregulation of TNFα typically stimulates expression of IL-10, which then downregulates TNFα to keep inflammation in check [34, 52]. Thus, while the early effect is to inhibit pro-inflammatory TNFα expression, and perhaps augment anti-inflammatory IL-10, later the result is that the entire cytokine response is suppressed. This feedback relationship is likely also responsible for the increases in both cytokines at 3 days after stroke in the Adrb2^cKO^ mice. Similar effects have been reported previously where neutralization of TNFα downregulates IL-10 [53] and in some cases, IL-10 and TNFα are downregulated by the same treatment [54].

The β2-adrenergic receptor signals through multiple complex pathways; for this reason, it is likely that its effect on inflammation depends on cell type and context of the inflammatory insult. The β2-adrenergic receptor is a 7-transmembrane receptor that is typically coupled to *G*_*s*_ proteins. Canonical stimulation of β2-adrenergic receptors expressed by immune cells increases intracellular cyclic-AMP and activates the protein kinase A pathway, generally resulting in reduced expression of pro-inflammatory factors such as TNFα and reactive oxygen species [7, 55–57]. However, the β2-adrenergic receptor is capable of signaling through multiple other pathways. When the β2-adrenergic receptor is phosphorylated, its coupling can switch from G_s_ to G_i_ which can actually have pro-inflammatory outcomes [58–60]. Additionally, β-arrestins can bind to the phosphorylated β2-adrenergic receptor, with effects including desensitization, internalization, or induction of signaling through the alternate ERK1/2 pathway [58, 61]. Here, we observed predominantly canonical immunosuppressive effects of β2-adrenergic receptor stimulation after stroke, although future work is needed to elucidate how β2-adrenergic signaling pathways in the brain are regulated over time.

Another factor to consider in future studies is that β2-adrenergic signaling may have particular effects on different cell types involved in the post-stroke inflammatory response. Both brain-resident microglia and MDMs infiltrating from the bloodstream have crucial, non-redundant roles in the neuroimmune response after stroke. A limitation of the present study is that we did not distinguish between these two cell types. In our clenbuterol experiment in which gene expression of TNFα was downregulated in brain tissue 4 h post-stroke, we can assume that these changes in inflammation are attributable to brain-resident cells, because blood-borne macrophages do not infiltrate the brain parenchyma at this early time point [16]. However, at 3 days post-stroke, we did not identify the cellular source of changes in inflammatory gene expression or distinguish between microglia or MDMs in the brain, since CD68 and Iba1 are markers known to label both cell types. Future experiments using cell-specific genetic markers might be useful for isolating these individual cell types and distinguishing the differential effects of β2-adrenergic activity. However, the two cell types likely respond analogously. Previous literature generally suggests that β2-adrenergic signaling affects microglia and peripheral macrophages similarly and results in similar effects on cytokines in both cell types to those we observed here [57, 62].

## Conclusion

This work shows that increasing β2-adrenergic receptor stimulation after ischemic stroke generally suppresses the neuroimmune response and leads to larger stroke volume. The specific effects of the β2-adrenergic receptor agonist clenbuterol include reduced blood pressure, enlarged morphology and reduced proliferation of microglia/MDMs, and reduced expression of both pro- and anti-inflammatory molecules in the brain. Selective knockout of the β2-adrenergic receptor from microglia and monocyte-lineage cells increased expression of similar pro- and anti-inflammatory mediators. Since β2-adrenergic receptor stimulation was not solely anti-inflammatory, we hypothesize that multiple complex signaling pathways underlie the effects of β2-adrenergic signaling on post-stroke inflammation. Given that outflow of the sympathetic nervous system may be increased after stroke and that many stroke patients are prescribed with β-blockers or sympathomimetic adrenergic agonists, this work highlights the importance of understanding the role of the β2-adrenergic signaling pathway on stroke pathophysiology and outcomes, and specifically that stimulating β2-adrenergic signaling in the subacute period after stroke may be harmful.

## Abbreviations

cAMP: Cyclic AMP
IL-10: Interleukin-10
IL-1β: Interleukin-1β
IL-6: Interleukin-6
iNOS: Inducible nitric oxide species
LPS: Lipopolysaccharide
MCAO: Middle cerebral artery occlusion
MDM: Monocyte-derived macrophage
TNFα: Tumor necrosis factor-α
